# Health insurance & responsiveness to communities & patients: The future of health systems in India

**Published:** 2011-01

**Authors:** D.H. Peters, B. Kanjilal

**Affiliations:** Health Systems Program Department of International Health Johns Hopkins Bloomberg School of Public Health Suite E8-132, 615 N. Wolfe St. Baltimore MD, USA 21205

Devadasan and colleagues^1^ in this issue highlight a number of important issues concerning the future of India’s health care system. At one level, the paper brings to the fore the important issues of providing adequate risk protection in health, the increasing need to focus on quality of health care, and to take patient and community perspectives into consideration. The paper also reveals the inter-connected but unpredictable relationships between health financing strategies and the supply and demand of health care.

The authors^1^ employ a study design that involves assessing patient satisfaction after a hospitalization for insured and uninsured patients, which limits the certainty of conclusions about the potential causal relationships between the use of community health insurance (CHI), patient satisfaction, and other aspects of quality of care. The design is susceptible to selection bias, small sample size, and the inability to test the effect of change in insurance status, all of which may contribute to the apparent lack of differences. Nonetheless, the absence of an association between satisfaction and insurance status among hospitalized patients in two well established CHI schemes in Tamil Nadu suggest that just because there are good theoretical reasons why health insurance should lead to higher patient satisfaction – because insured patients should be more reassured and empowered than the uninsured, because providers who are guaranteed payment may provide better services, or because payment can be linked to providing high quality of care – what happens in practice can be quite different. The authors point out that none of these potential theoretical advantages were actually realized in practice in the study areas. Such findings are consistent with research across a wide range of strategies intended to improve health services, and demonstrates that success in implementation is highly contextual^2^. Research on the implementation of health strategies suggests that involvement of patients and communities are important component of success of many strategies, along with engagement of other key stakeholders (*e.g*. health providers, government), and approaches that use data to continually revise strategies as these are implemented.

Patient satisfaction and perceptions of quality have not been reliably influenced by specific financing and health care interventions, as demonstrated by other studies in Asia. One quasi-experimental study in Uttar Pradesh introduced formal user fees and management reforms to improve quality of care^3^. Although the efforts did lead to increased overall patient satisfaction and improvements in objective measures of quality of care and increased utilization, there were significant differences in satisfaction between wealthy and poor populations, with improvements in patient satisfaction among the poor occurring only at the more peripheral levels of care (*i.e*. at community health centers rather than hospitals). In Afghanistan, one cross-sectional study found that factors related to patient interaction with the health provider (*e.g*. good communication, thoroughness of physical examination) were more important determinants of patient satisfaction than other structural features health care quality^4^, whereas a prospective controlled study also in Afghanistan found that different types of contracting with service providers had no effect on patient satisfaction^5^. By comparison, contracting with non-governmental organizations produced improved client satisfaction in Bangladesh^6^, but had a negative effect in Cambodia^7^.

Patient and community perceptions of health care provision and financing are increasingly important factors in a well functioning health care system. For example, patients and civil society organizations can provide practical roles to enhance regulation and accountability in a health system^8^. Improving patient perceptions is an important goal in itself, and also plays a pivotal role in influencing behaviours important to health. Gilson argues that health systems are intrinsically relational, and that trust is a relevant factor in several dimensions of health care^9^, including an important component of health worker performance^10^. Empiric work in Cambodia has shown that trust can also be a strong influence in villager’s willingness to enroll in CHI schemes^11^.

Providing protection from the financial risks of ill health is a growing priority to both reduce poverty and improve access to health care for Indians^12^. CHI is clearly not a panacea for all the health financing and delivery challenges in India. Although Devadasan and colleagues did not find a significant association between CHI enrollment and patient satisfaction in their study^1^, this does not mean that CHI is not worth pursuing. Rather, it raises the need to pay closer attention to how strategies are actually implemented, and to consider multiple perspectives and consequences when re-design programmes. It is also important to have more comprehensive intervention and evaluation approaches that can simultaneously consider supply and demand side factors, financing, incentives, and accountabilities. For a researcher, it points to the need for further experimentation and in- depth research, preferably prospective research that can consider these multiple dimensions of the health care system, and examine intended and unintended consequences. Greater understanding of the complex utility function of the users in developing countries - insured and uninsured – is also required to solve these puzzles, and adopt a quality-oriented CHI scheme. Devadasan and colleagues have provided a useful service in exploring the inter-dependencies of an important health financing initiative. The challenge is to continue innovation and research along this vein.
